# Necrostatin-1 Prevents Ferroptosis in a RIPK1- and IDO-Independent Manner in Hepatocellular Carcinoma

**DOI:** 10.3390/antiox10091347

**Published:** 2021-08-25

**Authors:** Hanna Yuk, Md Abdullah, Do-Hyung Kim, Haeseung Lee, Seung-Jin Lee

**Affiliations:** 1 College of Pharmacy, Chungnam National University, 99 Daehak-ro, Yuseong-gu, Daejeon 34134, Korea; yukhn94@naver.com (H.Y.); abdulpharm@o.cnu.ac.kr (M.A.); nicesoo86@nate.com (D.-H.K.); 2 College of Pharmacy, Pusan National University, Busan 46241, Korea; haeseung@pusan.ac.kr

**Keywords:** necrostatin-1, RIPK1, ferroptosis, system x_c_^−^, necroptosis, oxidative stress, lipid peroxidation, SLC7A11, bioinformatics

## Abstract

Ferroptosis is caused by the iron-mediated accumulation of lipid peroxidation, which is distinct from apoptosis and necroptosis. Necrostatin-1 inhibits receptor-interacting serine/threonine-protein kinase 1 (RIPK1) to initiate necroptosis; it also inhibits indoleamine 2,3-dioxygenase (IDO) to regulate tumor immunity. However, few studies have examined the off-target effect of necrostatin-1 on the ferroptosis pathway. The present study examined whether necrostatin-1 could interrupt ferroptosis induced by system xc- inhibitors (sulfasalazine and erastin) and a glutathione peroxidase 4 inhibitor (RSL3) in Huh7 and SK-HEP-1 cells. Necrostatin-1 completely prevented decreases in cell viability induced by sulfasalazine and erastin; it partially blunted decreases in cell viability induced by RSL3. Necrostatin-1, ferrostatin-1, and deferoxamine repressed sulfasalazine-provoked membrane permeabilization, as detected by 7-aminoactinomycin D staining and lipid peroxidation measured using a C11-BODIPY probe. However, other RIPK1 inhibitors (necrostatin-1s and GSK2982772) and an IDO inhibitor (1-methyl-D-tryptophan) did not recover the decrease in cell viability induced by sulfasalazine. Necrostatin-1 potentiated sulfasalazine-induced expression of xCT, a catalytic subunit of system xc- in these cells. These results demonstrated that necrostatin-1 blocked ferroptosis through a mechanism independent from RIPK1 and IDO inhibition in Huh7 and SK-HEP-1 cells, indicating that its antioxidant activity should be considered when using necrostatin-1 as a RIPK1 inhibitor.

## 1. Introduction

Regulated cell death can be classified according to mechanistic and essential processes. Ferroptosis is a recently defined form of regulated cell death characterized by the iron-dependent accumulation of fatal lipid-structured free radicals, which leads to membrane permeabilization [1]. Glutathione peroxidase 4 (GPX4) is essential for preventing the accumulation of toxic lipid-reactive oxygen species by reducing lipid peroxides into lipid alcohols via glutathione [2]. For glutathione synthesis, L-cysteine can be either synthesized de novo through the transsulfuration pathway or supplied by the reduction of L-cystine imported from extracellular space through system x_c_^−^ [3,4]. Some populations of cancer cells show epigenetic silencing or defects in the transsulfuration pathway; thus, they are exclusively dependent on L-cystine uptake through system x_c_^−^ [5]. GPX4 inhibitors (e.g., RSL3) and system x_c_^−^ inhibitors (e.g., erastin and sulfasalazine) promote ferroptosis, which can be prevented by lipophilic antioxidants, ferrostatin-1, or iron chelators [6,7,8,9].

Necroptosis is a form of non-apoptotic cell death linked to pathological conditions with an overt inflammatory signature, including ischemic brain injury, multiple sclerosis, Alzheimer’s disease, and Crohn’s disease [5]. Necroptosis is initiated by perturbations in the microenvironment detected by specific death receptors (e.g., Fas cell surface death receptor and tumor necrosis factor receptor 1) and pathogen recognition receptors (e.g., Toll-like receptors 3 and 4) [5]. In the presence of sufficient expression of receptor-interacting serine/threonine kinase 3 (RIPK3) and concomitant inhibition of pro-caspase 8 and FLIPL, death receptor ligands induce binding of RIPK1 to RIPK3 through an interaction between their respective RIP homotypic interaction motif domains [10]. Active RIPK3 catalyzes the phosphorylation of mixed lineage kinase domain-like protein, resulting in the formation of mixed lineage kinase domain-like protein oligomers that translocate to the plasma membrane. These oligomers directly act as a pore-forming complex in the cell membrane and indirectly disturb Ca^2+^ or Na^+^ ion channels, ultimately contributing to intracellular osmotic pressure and cell death.

Necrostatin-1 (5-(indol-3-ylmethyl)-3-methyl-2-thio-hydantoin) was identified as a small molecule that could inhibit necrotic cell death via tumor necrosis factor signaling through phenotypic screening [11]. Subsequently, necrostatin-1 was identified as an allosteric inhibitor of RIPK1 through in vitro kinase, homology modeling, and mutation analysis using recombinant RIPK1 [11,12]. Necrostatin-1 is identical to methyl-thiohydantoin-tryptophan, an inhibitor of the potent immunomodulatory enzyme indoleamine 2,3-dioxygenase (IDO), which is a rate-determining enzyme in tryptophan catabolism [13]. Therefore, inhibitory activity against IDO should be considered when interpreting the activity of necrostatin-1 [14,15]. Furthermore, necrostatin-1 exhibited partial inhibitory effects on PAK1 and PKAcα in an analysis of 98 human kinase activities [13]. Structurally analogous necrostatin-1s (7-Cl-O-Nec-1) and benzoxazepinone-structured GSK2982772 were reportedly more stable in body metabolism than necrostatin-1; they also had higher selectivity for RIPK1 [12,16].

In our previous study, a sulfasalazine-induced decrease in cell viability was prevented by ferrostatin-1, although it was not prevented by Z-VAD-FMK and chloroquine [17]. Necrostatin-1 also had a protective effect against these cell deaths. However, the mechanism by which necrostatin-1 interacts with the ferroptosis pathway is unknown. This study was designed to compare the effect of necrostatin-1 on cell death induced by sulfasalazine, erastin, or RSL3 with the effects of other pharmacological inhibitors of RIPK1 and IDO in Huh7 and SK-HEP-1 cell lines. In addition, this study evaluated the role of necrostatin-1 in the induction of antioxidant genes to elucidate the underlying mechanisms.

## 2. Materials and Methods

### 2.1. Materials

Huh7 and SK-HEP-1 cell lines were obtained from the Korean Cell Line Bank (Seoul, Korea). Dulbecco’s modified Eagle medium, RPMI-1640 medium, fetal bovine serum, L-glutamine, penicillin, and streptomycin were purchased from Thermo Fisher Scientific (Waltham, MA, USA). Sulfasalazine, ferrostatin-1, necrostatin-1, deferoxamine, and 1-methyl-D-tryptophan were purchased from Merck KGaA (Darmstadt, Germany). Erastin, RSL3, necrostatin-1s, and GSK2982772 and Z-VAD-FMK were obtained from Selleckchem (Houston, TX, USA). TNFα was supplied from PeproTech (Cranbury, NJ, USA) and SM164 was obtained from AdooQ Bioscience (Irvine, CA, USA).

### 2.2. Cell Culture

Huh7 cells were cultured in Dulbecco’s modified Eagle medium and SK-HEP-1 cells were grown in RPMI-1640 medium with 10% fetal bovine serum, 2 mM L-glutamine, 100 U/mL penicillin, and 100 µg/mL streptomycin at 37 °C in a humidified 5% CO_2_ incubator. Cells were regularly assessed to confirm the absence of mycoplasma contamination, as previously described.

### 2.3. Cell Viability

Cells were seeded in fresh medium in a 96-well plate at a density of 2000 cells per well. At 24 h after seeding, cells were exposed to ferrostatin-1, necrostatin-1, necrostatin-1s, GSK2982772, or 1-methyl-D-tryptophan, 1 h before dose-dependent treatment with sulfasalazine, erastin, or RSL3 for 24 h. Paired control cells were treated with dimethyl sulfoxide as a vehicle. Cell viability was measured by means of a CellTiter Glo^®^ assay, in accordance with the manufacturer’s instructions (Promega, Madison, WI, USA), using an EnVision^®^ multimode plate reader (Perkin Elmer, Waltham, MA, USA). IC_50_ value was calculated using the four parameters logistic curve equation using SigmaPlot 12.0 software (Systat, San Jose, CA, USA). Permeabilization of cell membrane was measured using the Guava^®^ Nexin reagent (Luminex, Austin, TX, USA), in accordance with the manufacturer’s instructions. Flow cytometry was performed using a Guava^®^ flow cytometer (Luminex, Austin, TX, USA) and analyzed with InCyte2.6 software (Luminex, Austin, TX, USA). For propidium iodide (PI) staining, cells were stained with 1 µg/mL PI for 30 min in the dark. Bright field and fluorescence images with excitation/emission at 535/617 nm were acquired using the EVOS™ FL Auto Imaging System (Thermo Fisher Scientific, Waltham, MA, USA). Three random images of each treatment group from independent experiments were captured to calculate the percentage of PI-positive cells.

### 2.4. Detection of Lipid Peroxidation

Lipid peroxide was detected using the Image-iT™ Lipid Peroxidation Kit, which is based on the lipophilic BODIPY^®^581⁄591 C11 probe (Thermo Fisher Scientific, Waltham, MA, USA). After treatment as indicated, 1 µM of the probe was added and the solution was incubated for 30 min at 37 °C. Cells were collected by trypsinization and the fluorescence from 5000 cells was measured with the excitation/emission set at 488/525 nm using a Guava^®^ easyCyte flow cytometer. Data were analyzed using InCyte2.6 software (Luminex, Austin, TX, USA).

### 2.5. Protein Sampling and Western Blot Analysis

Whole-cell lysates were prepared with lysis buffer and nuclear fraction was isolated with hypotonic buffer as described previously [18]. Protein concentrations were determined using the Bradford protein assay (Bio-Rad, Hercules, CA, USA), as previously described. Fifteen-microgram protein samples were then resolved using sodium dodecyl sulfate polyacrylamide gel electrophoresis on either 10% or 12% gels, transferred onto nitrocellulose membranes, probed with primary and secondary antibodies, and detected using a horseradish peroxidase substrate (Thermo Fisher Scientific, Waltham, MA, USA) via the iBright CL1000 Imaging System (Thermo Fisher Scientific, Waltham, MA, USA). Primary antibodies against xCT (#1269), RIP1 (#3493), phospho-RIP1 (Ser166)(#65746), RIP3 (#13526), phospho-RIP3 (Ser227)(#93654), MLKL (#14993), phospho-MLKL (Ser358)(#91689), and TXNRD1 (#15140) were purchased from Cell Signaling Technology (Danvers, MA, USA); antibodies against β-actin (sc-47778), heat shock protein 90 (sc-66048), lamin A/C (sc-6215), and Nrf2 (sc722) were obtained from Santa Cruz Biotechnology (Santa Cruz, CA, USA); an anti-GPX4 antibody (#ab125066) was supplied from Abcam (Cambridge, UK); an anti-IDO antibody (#654001) was purchased from BioLegend (San Diego, CA, USA). Horseradish peroxidase-linked secondary antibodies were obtained from Jackson ImmunoResearch (West Grove, PA, USA). All antibodies were diluted to between 1:2000 and 1:10,000.

### 2.6. Necrostatin-1-Induced Transcriptome Data

Genome-wide expression changes in various human cell lines by necrostatin-1 treatment were obtained from the GEO (Accession: GSE92742). We used the replicate-collapsed-moderated z-score (MODZ, level5 data) representing the difference in gene expression induced by necrostatin-1 compared to the corresponding controls. Z-score profiles with poor reproducibility (distil_cc_q75′ < 0.2 and pct_self_rank_q25 > 0.05) were filtered out. R software and the R package ‘cmapR’ was used to access and manipulate a GCTX file.

### 2.7. Statistical Analyses

Data are expressed as means ± standard deviations (SD), with n indicating the number of independent in vitro experiments for a particular analysis. *p* < 0.05 was considered to indicate statistical significance. Statistical analyses were performed using one-way analysis of variance, followed by Tukey’s multiple-comparison test. All statistical analyses were conducted using SPSS Statistics for Windows (version 26; SPSS Inc., Chicago, IL, USA).

## 3. Results

### 3.1. Effect of Necrostatin-1 on Changes in Cell Viability Induced by Sulfasalazine, Erastin, and RSL3

**Figure 1:**
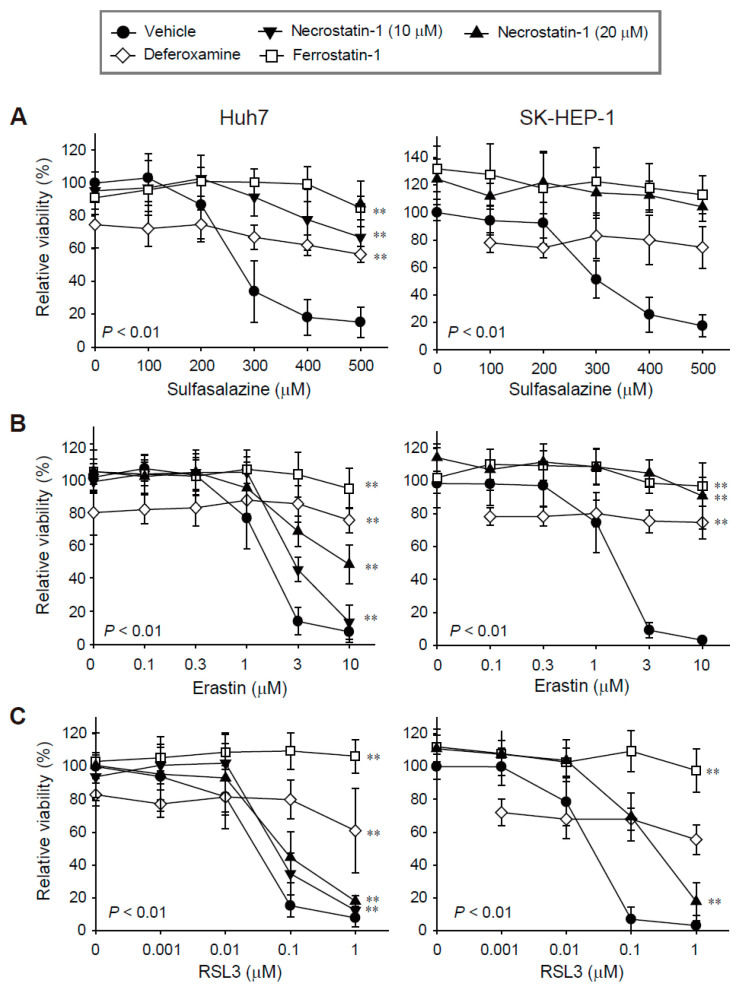
Decreases in cell viability induced by sulfasalazine, erastin, or RSL3 were prevented by pre-treatment with necrostatin-1, deferoxamine, or ferrostatin-1. Huh7 (left) and SK-HEP-1 (right) cells were treated with vehicle (circle), necrostatin-1 (inverted triangle for 10 μM; triangle for 20 μM), 100 μM deferoxamine (iron chelator, diamond), or 20 μM ferrostatin-1 (lipophilic antioxidant, square), for 1 h. Subsequently, sulfasalazine (**A**), erastin (**B**), or RSL3 (**C**) was applied for 24 h in a dose-dependent manner; cell viability was measured with a CellTiter-Glo^®^(Promega, Madison, WI, USA) assay. Each experiment was repeated 3–6 times in triplicate. Data are expressed as means ± SDs. ** *p* < 0.01, vehicle-treated versus inhibitor-treated.

We evaluated the effects of necrostatin-1 on ferroptosis in Huh7 and SK-HEP-1 cells because we found these cell lines to be highly sensitive to ferroptosis in our previous study [17]. Treatment with sulfasalazine, erastin, and RSL3 for 24 h decreased cell viability in a dose-dependent manner. The IC_50_ values in Huh7 and SK-HEP-1 cells were 250.95 μM and 288.26 μM for sulfasalazine, 1.33 μM and 1.38 μM for erastin, and 0.03 μM and 0.02 μM for RSL3, respectively (Figure 1).

Ferrostatin-1 completely protected against growth inhibition induced by sulfasalazine, erastin, and RSL3 in the Huh7 and SK-HEP-1 cells, as expected. While the application of 100 μM deferoxamine alone reduced cell viability by 20%, it significantly protected against sulfasalazine-, erastin-, and RSL3-induced growth inhibition. Necrostatin-1 reversed sulfasalazine-induced growth inhibition; the protective effects of necrostatin-1 and ferrostatin-1 at 20 μM were similar in Huh7 and SK-HEP-1 cells. Necrostatin-1 prevented erastin-induced growth inhibition in Huh7 cells in a dose-dependent manner. Growth retardation induced by 10 μM erastin was reversed by 43.6% in Huh7 cells, whereas the effect of erastin was completely reversed in SK-HEP-1 cells. Necrostatin-1 at 20 μM rescued the decrease in cell viability induced by 0.1 μM RSL3 treatment by 34.7% in Huh7 cells and by 67.1% in SK-HEP-1 cells. Necrostatin-1 did not prevent the decrease in cell viability induced by 1 μM RSL3 in these cell lines. In summary, necrostatin-1 significantly blocked the decrease in cell viability induced by sulfasalazine and erastin in both cell lines; it partially reversed the reduction in cell viability caused by RSL3 in SK-HEP-1 cells.

### 3.2. Effect of Necrostatin-1 on Increases in Membrane Permeability and Lipid Peroxidation Induced by Sulfasalazine

**Figure 2:**
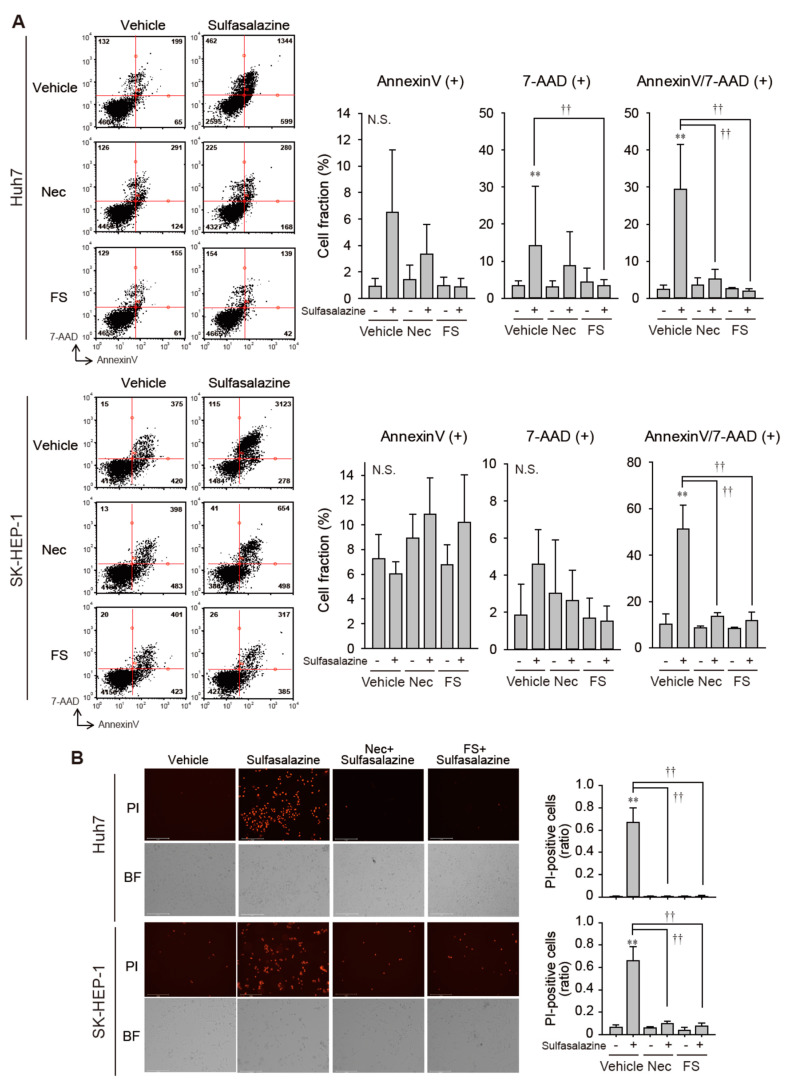
Necrostatin-1 rescued cells from sulfasalazine-induced membrane permeabilization. (**A**) After treatment with 20 μM necrostatin-1 (Nec) or 20 μM ferrostatin-1 (FS) for 1 h, vehicle or 500 μM sulfasalazine were applied to Huh7 and SK-HEP-1 cells for 24 h. Cells were sampled for staining with annexin-V and 7-AAD, then examined via flow cytometry. (**B**) Vehicle or 500 μM sulfasalazine were treated to Huh7 and SK-HEP-1 cells for 24 h after exposure of 20 μM Nec or 20 μM FS for 1 h. Cells were stained with 1 µg/mL PI and examined to obtain bright field (BF) and PI image. Representative images (left) are shown for selected groups. Each experiment was repeated 4 times and data are expressed as means ± SDs. ** *p* < 0.01, vehicle versus sulfasalazine; ^††^ *p* < 0.01, sulfasalazine versus sulfasalazine with inhibitor; N.S., not significant.

Because necrostatin-1 effectively protected against growth inhibition induced by sulfasalazine, we tested whether necrostatin-1 would prevent the increase in membrane permeability and lipid peroxidation, which is a characteristic of ferroptosis [1]. In Huh7 cells, 24 h of treatment with 500 μM sulfasalazine increased the annexin V-positive cell fraction to 6.5% (*p* < 0.05) and the fraction of annexin-V/7-AAD-positive cells to 29.5% (*p* < 0.01) (Figure 2A upper). Necrostatin-1 significantly reduced the levels of annexin-V/7-AAD-positive cells. In SK-HEP-1 cells, exposure to 500 μM sulfasalazine for 18 h resulted in the increase of annexin-V/7-AAD-positive cell fraction to 51.4% (*p* < 0.01) (Figure 2A lower). Levels of annexin-V/7-AAD-positive cells in the necrostatin-1- and ferrostatin-1-pretreated groups were similar to corresponding levels in the control group.

We confirmed whether necrostatin-1 could prevent sulfasalazine-induced cell death by PI staining assay (Figure 2B). Sulfasalazine increased the PI-positive cell fraction to 67% in Huh7 cells and 66% in SK-HEP-1 cells. Pretreatment of necrostatin-1 and ferrostatin completely blunted the increase of propidium-positive cells in both cell lines.

**Figure 3:**
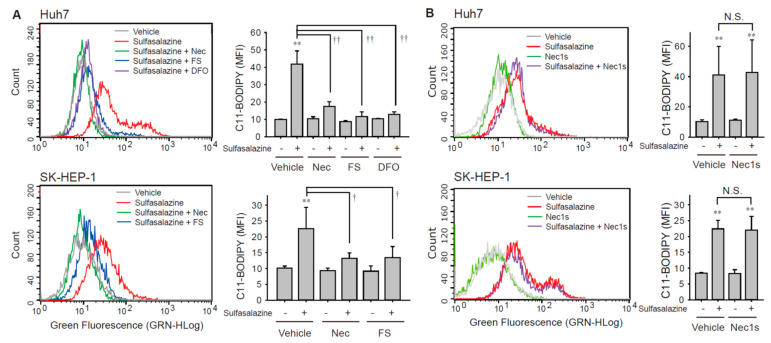
Necrostatin-1 blocked sulfasalazine-induced accumulation of lipid peroxidation. (**A**) Vehicle or 500 μM sulfasalazine were applied to Huh7 cells for 18 h or to SK-HEP-1 cells for 24 h after treatment with 20 μM necrostatin-1 (Nec), 20 μM ferrostatin-1 (FS), or 100 μM deferoxamine (DFO) for 1 h (*n* = 3). (**B**) After pretreatment of 20 μM necrostatin-1s (Nec1s), cells were exposed to sulfasalazine as described in (A) in Huh7 cells (*n* = 4) or SK-HEP-1 cells (*n* = 3). Cells were sampled for staining with a C11-BODIPY probe and examined via flow cytometry. Representative images with selected groups are shown to avoid complication. Each experiment was independently repeated and data are expressed as means fluorescence intensity (MFI) ± SDs. ** *p* < 0.01, vehicle versus sulfasalazine; ^†^ *p* < 0.05, ^††^ *p* < 0.01, sulfasalazine versus sulfasalazine with inhibitor; N.S., not significant.

Next, we investigated whether the sulfasalazine-induced increase in the accumulation of lipid peroxide was affected by necrostatin-1 using the C11-BODIPY probe, which is a lipid peroxidation sensor. The application of 500 μM of sulfasalazine for 18 h increased lipid peroxidation by 4.2-fold in Huh7 cells (*p* < 0.01) and 2.2-fold (*p* < 0.01) in SK-HEP-1 cells (Figure 3A). This sulfasalazine-induced increase in lipid peroxidation was reduced by 94.8% and 90.5% by deferoxamine and ferrostatin-1, respectively, in Huh7 cells; it was reduced by 73.5% by ferrostatin-1 in SK-HEP-1 cells. Consistently, necrostatin-1 significantly decreased sulfasalazine-induced lipid peroxidation by 75.9% in Huh7 cells and 76.1% in SK-HEP-1 cells. In contrast, necrostatin-1s failed to prevent the accumulation of lipid peroxidation provoked by sulfasalazine in Huh7 cells and SK-HEP-1 cells (Figure 3B).

### 3.3. RIPK1 and IDO Were Not Involved in Sulfasalazine-Induced Reduction in Cell Viability

**Figure 4:**
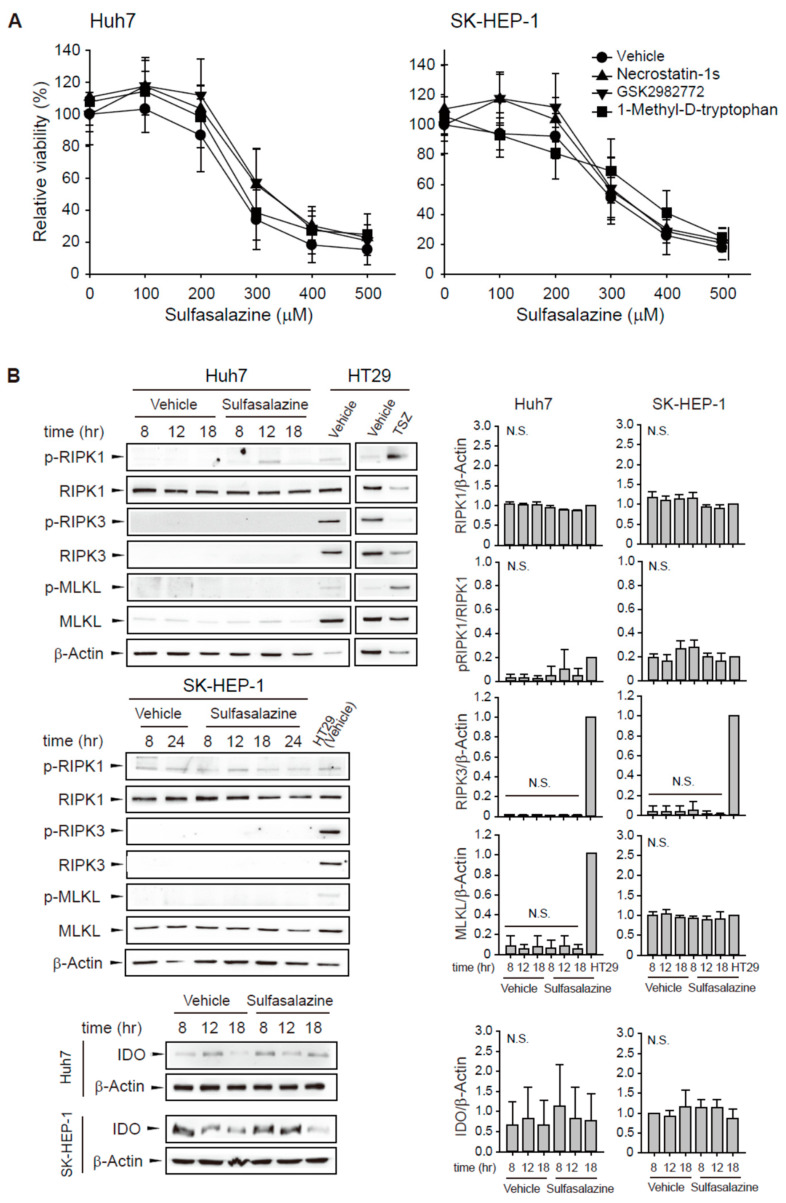
Other RIPK1 inhibitors and IDO inhibitor did not prevent the decrease in cell viability induced by sulfasalazine. (**A**) Huh7 and SK-HEP-1 cells were treated with vehicle (circle) or 20 μM necrostatin-1s (RIPK1 inhibitor, triangle), 20 μM GSK2982772 (RIPK1 inhibitor, inverted triangle), or 500 μM 1-methyl-D-tryptophan (IDO inhibitor, square) for 1 h, followed by sulfasalazine in a dose-dependent manner for 24 h. Cell viability was measured with a CellTiter-Glo^®^ assay. (**B**) Cells were treated with 500 μM sulfasalazine for indicated periods and subjected to Western blot analysis. HT29 cells treated with vehicle or TSZ (50 ng/ml TNFα, 1 μM SM164 was 20 μM Z-VAD-FMK) for 12 h as a positive necroptosis model. Relative expression was calculated in compared with vehicle (8 h) group for IDO or HT29 (vehicle) group for others. Each experiment was repeated 3 times in triplicate and data are expressed as means ± SDs. N.S., not significant.

To evaluate whether other RIPK1 inhibitors would exert a protective effect similar to necrostatin-1, we tested two RIPK1 selective inhibitors: structural analogous necrostatin-1s and benzodiazepinone-structured GSK2982772. The decrease in cell viability induced by sulfasalazine was unchanged by pre-treatment with 20 μM necrostatin-1s or 20 μM GSK2982772 in Huh7 and SK-HEP-1 cells (Figure 4A). Because necrostatin-1 has an inhibitory effect on IDO, we examined the effect of 1-methyl-D-tryptophan, another IDO inhibitor. We found that pretreatment with 500 μM 1-methyl-D-tryptophan did not rescue the sulfasalazine-induced decrease in cell viability.

Next, sulfasalazine-induced changes in RIPK1/RIPK3/MLKL activation and IDO expression were examined (Figure 4B). Phosphorylation of RIPK1 at serine 166, RIPK3 at serine 227, and MLKL at serine 358 plays a role for the activation of necroptosis [19,20]. HT29 cells were used as a positive necroptosis model which express RIPK1, RIPK3, and MLKL and activate necroptosis in response to TSZ (TNFα/SM164 /Z-VAD-FMK) [21]. Huh7 and SK-HEP-1 cells expressed RIPK1 protein, but sulfasalazine did not significantly increase RIPK1 phosphorylation. Huh7 and SK-HEP-1 cells barely expressed RIPK3 in comparison with HT29 cells. MLKL was expressed in SK-HEP-1 cells, but it was not activated by sulfasalazine. IDO expression was very low in Huh7 cells and not significantly changed by sulfasalazine in both cell lines. All of these results indicate that the protective effect of necrostatin-1 on sulfasalazine-induced ferroptosis was not the result of RIPK1 or IDO inhibition in these cells.

### 3.4. Changes in the Expression of TXNRD1, GPX4, and xCT Induced by Sulfasalazine and/or Necrostatin-1

**Figure 5:**
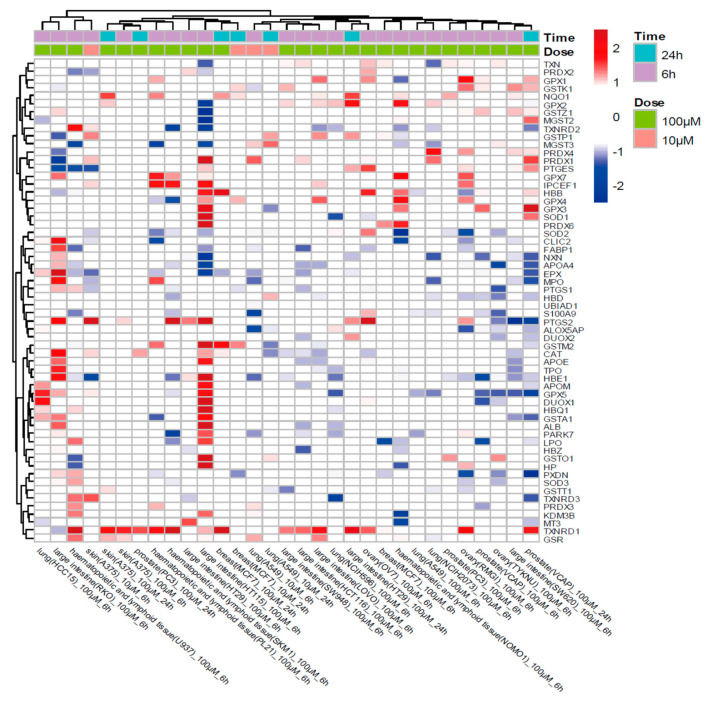
Connectivity Map data of necrostatin-1. Necrostatin-1-induced expression changes in 60 genes related to antioxidant activity. The antioxidant genes (GO:0016209) were collected from Gene Ontology Resource (http://geneontology.org/; accessed on 27 January 2021).

To elucidate the underlying mechanism of necrostatin-1 application for ferroptosis protection, we investigated necrostatin-1-induced gene expression changes of antioxidant genes using the Connectivity Map data [22]. This database provides transcriptomic profiles of 55 human cell lines treated with 10 or 100 μM necrostatin-1, but it does not include the transcriptomes of other RIPK1 or IDO inhibitors. Of the 60 genes involved in antioxidant activity (GO:0016209), TXNRD1 was the most frequently overexpressed gene across various cell lines: 15/31 cases of cancer cell lines showed upregulation of TXNRD1 (z-score > 1) by 100 μM necrostatin-1 (Figure 5).

**Figure 6:**
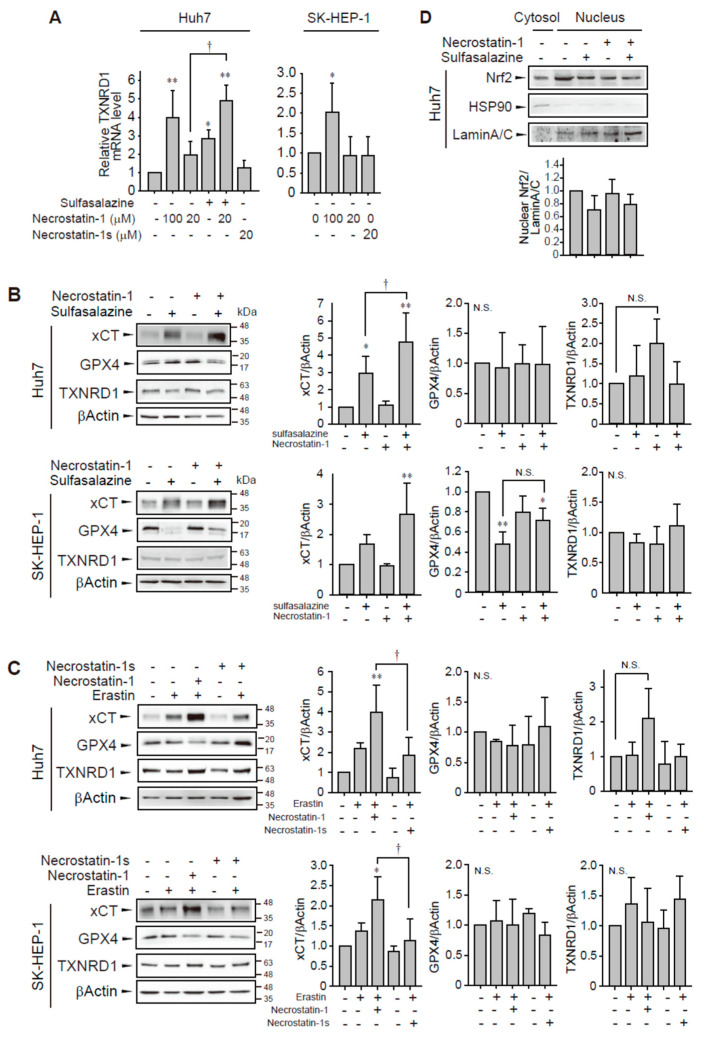
Effect of necrostatin-1 on xCT, GPX4, and TXNRD1 expression. (**A**) Huh7 cells were treated with vehicle, 20 μM or 100 μM necrostatin-1 or 20 μM necrostatin-1s for 1 h, followed by 500 μM sulfasalazine for 24 h. SK-HEP-1 cells were incubated with necrostatin-1 or necrostatin-1s for 24 h. Cells were lysed and subjected to real-time qPCR. Western blot analysis was performed with the cells which were pre-treated with 20 μM necrostatin-1 or 20 μM necrostatin-1s for 1 h and subsequently exposed to 500 μM sulfasalazine (**B**) or 3 μM erastin (**C**) for 24 h. (**D**) Cells were exposed to 500 μM sulfasalazine for 18 h after pre-treatment of 20 μM necrostatin-1 and fractionated as cytosol and nucleus. Heat shock protein 90 (HSP90) and lamin A/C were detected as a cytosolic and nuclear marker, respectively. Relative expression was calculated in comparison with vehicle group. Each experiment was repeated 3 times and data are expressed as means ± SDs. * *p* < 0.05, ** *p* < 0.01, vehicle versus treatment; ^†^ *p* < 0.05, necrostatin-1 versus sulfasalazine with necrostatin-1; N.S., not significant.

In a confirmation study, we found that the application of 100 μM necrostatin-1 increased TXNRD1 mRNA expression in Huh7 and SK-HEP-1 cells (Figure 6A). However, necrostatin-1 and necrostatin-1s had no significant effect on TXNRD1 mRNA expression at 20 μM in these cells. The application of 500 μM sulfasalazine increased in Huh7 cells and this increase was enhanced by pretreatment with 20 μM necrostatin-1.

Because GPX4 and system x_c_^−^ are key regulators of ferroptosis, we measured the expression levels of TXNRD, GPX4, and xCT which is a catalytic subunit of system x_c_^−^. Although sulfasalazine inhibits system x_c_^−^ activity, sulfasalazine-treated cells showed increased expression of xCT in Huh7 cells (Figure 6B). Pretreatment of necrostatin-1 and subsequent exposure of sulfasalazine enhanced the expression of xCT in both cell lines, suggesting that necrostatin-1 may contribute to the enhanced antioxidant capacity in response to sulfasalazine. GPX4 expression did not significantly change following necrostatin-1 and/or sulfasalazine treatment in Huh7 cells. Sulfasalazine decreased GPX4 expression in SK-HEP-1 cells. Degradation of GPX4 by sulfasalazine has been also reported in breast and pancreatic cancer cells [23,24]. The change in GPX4 expression by sulfasalazine was not significantly changed by necrostatin-1 pre-treatment in SK-HEP-1 cells. In contrast to the changes in mRNA expression, the protein level of TXNRD1 was not significantly changed following treatment with 500 μM sulfasalazine and/or 20 μM necrostatin-1.

Next, we examined whether necrostatin-1 would also enhance erastin-mediated xCT expression and compared the effects of necrostatin-1 with necrostatin-1s (Figure 6C). Treatment of necrostatin-1 with erastin significantly enhanced xCT expression in Huh7 and SK-HEP-1 cells. In contrast, pretreatment of necrostatin-1s did not increase xCT expression. GPX4 expression was not significantly changed by erastin, and/or necrostatin-1 treatment. The increases of TXNRD1 protein expression by necrostatin-1 with erastin was marginal and statistically insignificant. Therefore, while necrostatin-1 could elicit transcriptional activation of TXNRD1, this response is unlikely to be sufficient for induction of translational activation in these cells.

In order to examine the mechanism of necrostatin-1 to enhance xCT expression, we examined the nuclear localization of nuclear factor erythroid 2–related factor 2 (Nrf2) which is a transcription factor to bind antioxidant response element of xCT promoter [25]. However, treatment of sulfasalazine and/or necrostatin-1 for 18 h had no effect on the distribution of Nrf2 in Huh7 cells. Necrostatin-1 might involve other transcriptional or post-transcriptional process for xCT induction in these cells. Taken together, these data indicate that the enhancement of xCT expression by necrostatin-1 in the presence of sulfasalazine or erastin could be a mechanism for protection against ferroptosis in Huh7 and SK-HEP-1 cells.

## 4. Discussion

Necrostatin-1 is a representative necroptosis inhibitor that directly binds to RIPK1 and represses the autophosphorylation of RIPK1. Despite concerns regarding the interpretation of results obtained using necrostatin-1 [14], it continues to be widely employed in ferroptosis research to discriminate ferroptosis from necroptosis. Necrostatin-1, unlikely to lipophilic antioxidant, failed to inhibit lipid peroxidation and ferroptosis in other studies using various cell lines [1,8]. In contrast to these previous studies, our current findings demonstrate that necrostatin-1 significantly protected against ferroptosis induced by sulfasalazine and erastin in Huh7 and SK-HEP-1 cell lines. This protective effect of necrostatin-1 against RSL3-induced ferroptosis was partial and cell-type specific. Necrostatin-1s, GSK2982772, and 1-methyl-D-tryptophan did not block the decrease in cell viability induced by sulfasalazine, indicating that necrostatin-1 could inhibit ferroptotic cell death in a RIPK1- and IDO-independent manner in some populations of cancer cells.

A previous study with necrostatin-1 derivatives showed that the protective effect of necrostatin-1 might involve mechanisms other than RIPK1 or IDO-1 inhibition [13]. Necrostatin-1i, an inactive variant of necrostatin-1 for RIPK1 inhibition, can inhibit IDO, whereas necrostatin-1s selectively inhibits RIPK1 alone (i.e., not IDO-1) [11]. Although the activity of necrostatin-1 derivatives against RIPK1 in vitro was strongly correlated with the inhibition of necroptosis in a Jurkat cell model [14], necrostatin-1, necrostatin-1i, and necrostatin-1s equally prevented tumor necrosis factor-induced mortality in mice at high doses. Furthermore, low doses of necrostatin-1 and necrostatin-1i were found to sensitize mice to tumor necrosis factor-induced mortality. Furthermore, necrostatin-1 protected SH-SY5Y cells from MPP^+^-induced death that had some features of ferroptosis, independently of RIPK1/RIPK3 [26]. Necrostatin-1 inhibited MPP^+^-induced cell death and lipid peroxidation, as well as RSL3-induced ferroptosis, in this cell line. Taken together, these data indicate that necrostatin-1 may interact with the cell death pathway through mechanisms unrelated to RIPK1 or IDO-1 inhibition in a cell context-dependent or cell death model-dependent manner. However, the conditions in which necrostatin-1 interacts with the ferroptosis pathway are unclear.

In this study, we found that necrostatin-1 could significantly inhibit ferroptotic cell death induced by sulfasalazine in Huh7 and SK-HEP-1 cells. These effects were not observed for other RIPK inhibitors (Figure 4A). Sulfasalazine did not activate RIPK1 and MLKL phosphorylation and RIPK3 protein expression was not detected in our cell models. Najafov A et al. [27] suggested that escape from necroptosis is prevalent across cancer types with an incidence rate of 83% through analysis with 941 cancer cell types and loss of RIPK3 is the primary factor correlating with the escape. Therefore, sulfasalazine is not likely to activate necroptosis in our cell models.

We attempted to infer the mechanisms of necrostatin-1 from a public Connectivity Map [22]. The results indicated that TXNRD1 could be a downstream gene of necrostatin-1 across cell lines. We confirmed that 100 μM necrostatin-1 induced the production of TXNRD1 mRNA and found that 20 μM necrostatin-1 potentiated TXNRD1 expression in combination with sulfasalazine. TXNRD1 protein expression by 20 μM necrostatin-1 with sulfasalazine or erastin tend to be increased, but the changes were marginal and not significant in Huh7 cells. The correlation and variation between mRNA and protein expression has been discussed in several literatures. Recent studies with high quality datasets suggest mRNA-protein correlation at gene-to-gene level is above 0.5 [28,29,30]. In general, protein concentrations can be largely determined by transcript levels at least on the bulk dataset and for steady-state conditions, but highly dynamic phases may cause stronger deviations from an ideal correlation via post-transcriptional regulation [28]. As we monitored the TXNRD1 expression during stress response of ferroptosis and TXNRD1 is a selenoprotein regulated by post-transcriptional effect [31], post-transcriptional process could play a role in temporal adaptation of TXNRD1 protein expression in our models.

When we explored the possibility that necrostatin-1 activates other antioxidant genes, we found that necrostatin-1 potentiated the protein expression of xCT with sulfasalazine or erastin. While sulfasalazine inhibits system x_c_^−^, it can induce xCT expression, perhaps as a compensatory mechanism for cell death. The enhancement of xCT expression by necrostatin-1 could overcome the inhibitory effect of sulfasalazine on system x_c_^−^. This mechanism supports the preferential protection of necrostatin-1 for xCT inhibition, rather than GPX4 inhibition (Figure 1). Although necrostatin-1 induced the expression of Nrf2 and heme oxygenase-1 in acute lung injury in mice [32], we did not observe activation of Nrf2 by necrostatin-1 in our cell models (Figure 6D). xCT expression is transcriptionally regulated by ATF4, STAT3/5, or p53 in addition to Nrf2 and stabilized by EGFR, CD44v, and the deubiquitylase OTUB1 [25]. Thus, these mechanisms might be involved in the potentiation of xCT expression by necrostatin-1 in Huh7 and SK-HEP-1 cell models.

## 5. Conclusions

Necrostatin-1 can inhibit ferroptotic cell death provoked by system x_c_^−^ inhibition in Huh7 and SK-HEP-1 cells, likely through the induction of xCT expression. Because necrostatin-1 could activate survival pathways shared with ferroptosis in a context-dependent manner, the use of necrostatin-1 as a RIPK1 inhibitor should accompany other selective RIPK1 inhibitor or RIPK1 knockdown. Notably, RIPK inhibition is an attractive pipeline for autoimmune processes and neurodegeneration. Our data showed that necrostatin-1 has structural uniqueness sufficient to protect against ferroptotic cell death, unlike necrostatin-1s and GSK2982772. Further studies to identify the antioxidant mechanisms as well as to determine the relevant structure-activity relationships would facilitate the development of a potent RIPK1 inhibitor with antioxidant activity.

## Data Availability

Data is contained within the article. Data used in Figure 5 is publically accessible at https://clue.io/cmap, accessed on 10 July 2021.
